# Long-Term Adherence to the Mediterranean Diet Reduces 20-Year Diabetes Incidence: The ATTICA Cohort Study (2002–2022)

**DOI:** 10.3390/metabo14040182

**Published:** 2024-03-25

**Authors:** Ioanna Kechagia, Thomas Tsiampalis, Evangelia Damigou, Fotios Barkas, Georgia Anastasiou, Evrydiki Kravvariti, Evangelos Liberopoulos, Petros P. Sfikakis, Christina Chrysohoou, Costas Tsioufis, Christos Pitsavos, Demosthenes Panagiotakos

**Affiliations:** 1 Department of Nutrition and Dietetics, School of Health Sciences & Education, Harokopio University, 17676 Athens, Greece; 2 Department of Clinical Dietetics-Nutrition, HYGEIA Hospital, 15123 Athens, Greece; 3 Department of Internal Medicine, Medical School, University of Ioannina, 45500 Ioannina, Greece; 4 First Department of Propaedeutic Internal Medicine, Medical School, National and Kapodistrian University of Athens, Laiko General Hospital, 15772 Athens, Greece; 5 First Cardiology Clinic, Medical School, National and Kapodistrian University of Athens, Hippokration Hospital, 11527 Athens, Greece

**Keywords:** mediterranean diet, type II diabetes mellitus, diabetes incidence, longitudinal, mediterranean diet trajectories, dietary patterns, prospective study, risk

## Abstract

This study aimed to evaluate the association between adherence to the Mediterranean diet and the 20-year incidence of type II diabetes mellitus (T2DM) among adults from the ATTICA study. This study involved a prospective cohort of 3042 men and women recruited at baseline from the Attica region in Greece. Sociodemographic, anthropometric, lifestyle, and clinical characteristics were evaluated at baseline and follow-up examinations; adherence to the Mediterranean diet was assessed through the MedDietScore (range 0–55); four Mediterranean diet trajectories were identified (i.e., increasing, decreasing, and sustained high and sustained low adherence levels). For the present analysis, data from 2000 individuals with complete information were used (age 43 ± 13 years; 49% men). Over the 20-year period, 26.3% (95%CI 24.4%, 28.3%) of participants developed T2DM; men exhibited a 1.5-times higher incidence compared to women (*p* < 0.001). Individuals consistently close to the Mediterranean diet throughout the studied period had an improved glycemic and lipidemic profile (at baseline and at 10-y follow-up) (all *p*-values < 0.001) and showed a 21% reduction in their 20-year risk of developing T2DM compared to those who were consistently away (RR = 0.79, 95%CI 0.47, 0.86). A long-term adherence to the Mediterranean diet is protective against the onset of T2DM and, therefore, could be incorporated in public health actions for the prevention of the disease.

## 1. Introduction

Diabetes is one of the fastest growing global health emergencies of the 21st century. More than a half billion people worldwide aged 20–79 years are currently living with this condition, i.e., 10.5% of the world’s population. The projections of the global prevalence of diabetes in the 20–79 age group indicate an increase of 46% from 2021 to 2046, with catastrophic socio-economic consequences given that the direct health expenditure will exceed USD one trillion by 2030 [1]. Diabetes-related mortality has also increased, given that 6.7 million people died during 2021 from diabetes-related causes [1], including both macrovascular (i.e., cardiovascular diseases) and microvascular (i.e., diabetic neuropathy, diabetic kidney disease, and diabetes-related eye disease) causes [2]. Therefore, there is an urgent need for nutritional recommendations concerning the primary prevention of diabetes, which is one of the four major non-communicable diseases [3].

The cornerstone of the primary prevention of diabetes is adopting a healthy lifestyle, including healthy body weight, healthy eating patterns, and avoiding smoking and harmful alcohol drinking, according to findings from large randomized controlled trials [4,5,6]. Amongst the so-called “healthy” diets, adherence to the Mediterranean diet is the most widely studied dietary pattern and it has been associated with a reduction in cardiometabolic disease incidence [7,8]. However, the effect of adherence to the Mediterranean diet on diabetes incidence is not well studied nor well appreciated; some investigators have underlined its long-lasting benefits on glycemic control [9], whereas others have failed to show any relationship, which is potentially due to the alarming westernization of lifestyle and the significant observed decline in Mediterranean diet adherence the last decades [10]. Thus, the purpose of the present work was to evaluate the association between the long-term trajectories of adherence to the Mediterranean diet and the 20-year incidence of type II diabetes mellitus (T2DM) in the sample of the ATTICA study, along with the implication of possible biological mechanisms that may be involved in the pathogenesis of T2DM.

## 2. Materials and Methods

### 2.1. Study Design

The ATTICA study is a prospective cohort that was carried out during 2001–2002. The participants of the study were from the Attica region (3.8 million population, containing 78% urban municipalities), which includes Athens, the capital city of Greece (3.15 million population). Three follow-up examinations were performed, i.e., 2006: 5-year follow-up; 2012: 10-year follow-up; 2022: 20-year follow-up. The main purpose of this study was to evaluate the distribution of a variety of lifestyle, sociodemographic, clinical, biochemical, and psychological risk factors at various time points and their association with long-term cardiometabolic disease incidence, including diabetes, as well as to assess the trajectories of these factors regarding their predictive significance. Additionally, information concerning the study aims, design, sampling procedure, and methodology exists in previously published papers [11].

#### Bioethics

The ATTICA study adheres to the ethical guidelines of the Declaration of Helsinki [12] and has been approved by the Ethics Committee of the First Cardiology Department of the National and Kapodistrian University of Athens (#017/01.05.2001), as well as the Ethics Committee of the Harokopio University (#38/29.03.2022). All participants were informed about the objectives and procedures of the study, and they provided their written consent to participate.

### 2.2. Sample Characteristics

The exclusion criteria concerning the participation were as follows: history of cardiovascular disease (CVD) or other atherosclerotic disease, chronic viral infections, and living in institutions. The sampling was random, and was stratified by sex, age group, and region, according to the 2001 census. In particular, out of the 4056 individuals that were initially invited, 3042 agreed to participate in the study (75% participation rate); 1514 were men (aged 46 ± 13 years; 18–87 years) and 1528 were women (aged 45 ± 13 years; 18–89 years). The evaluation of the participants took place through a standardized protocol at their homes or workplaces through face-to-face interviews by a team of health professionals, including cardiologists, nurses, dietitians, and general practitioners.

### 2.3. Baseline Evaluation

Participants were evaluated at baseline regarding their dietary and lifestyle habits, anthropometric indices, medical status, and cardiometabolic parameters. A detailed questionnaire was used to record their current medication and personal and family medical history, along with several sociodemographic characteristics.

Blood pressure, blood lipids, as well as anthropometric indices, i.e., body weight, height, waist circumference (WC), and hip circumference, were measured in all participants by the physicians of the study. Arterial blood pressure was measured three times with participants in a sitting position after a 30-min rest. Hypertension was defined as a utilization of antihypertensive drugs, an average systolic blood pressure (SBP) ≥ 140 mmHg, or an average diastolic blood pressure (DBP) ≥ 90 mmHg. Biochemical markers, i.e., total cholesterol (TC), high-density lipoprotein cholesterol (HDL-C), triglycerides (TGs), apoB100 (apolipoprotein B100), fasting glucose, fasting insulin, C-reactive protein, interleukin-6, and tumor necrosis factor-alpha were, measured in 12-h fasting blood samples using appropriate methods. Low-density lipoprotein cholesterol (LDL-C) was calculated according to the Friedewald formula. Hypercholesterolemia was defined as total cholesterol (TC) ≥ 200 mg/dL and/or the use of lipid-lowering agents, while T2DM was defined as fasting glucose ≥ 125 mg/dL or the use of antidiabetic drugs and/or insulin [13]. The measurements of body weight (in kilograms), height (in m), and waist (in cm) and hip (in cm) circumference were carried out following a standardized protocol [14]. Body Mass Index (BMI) was calculated as weight/height^2^ (in Kg/m^2^), and this was used to evaluate weight status in combination with waist and hip circumferences [15].

Dietary assessment was conducted via a validated semi-quantitative food frequency questionnaire, and habitual food intake was expressed as servings per day or week [16]. MedDietScore, an a priori diet index, was used to evaluate the adherence to a Mediterranean-type diet [17]. The range of the MedDietScore is between 0 and 55 points; higher points indicate a higher adherence to the traditional Mediterranean diet. The MedDietScore consists of 11 components. Mediterranean diet trajectories were also calculated to evaluate longitudinal adherence (2002–2022) to the traditional dietary pattern. Four Mediterranean diet trajectories were formed regarding the longitudinal change in the level of adherence, i.e., increasing adherence level from low adherence at baseline examination (i.e., MedDietScore below the median value of 27) to high adherence at 10-year follow-up examination (i.e., MedDietScore ≥ 27); decreasing adherence level (from high baseline adherence to low 10-year adherence); sustained high adherence level (high adherence at both time points); and sustained low adherence level (low adherence at both time points). The short-form International Physical Activity Questionnaire (IPAQ), which was validated for the Greek population, was used to assess individuals’ physical activity level regarding frequency (times weekly), duration (in minutes per time), and intensity (expressed as calories per time) over a typical week [18]. The category “current smokers” included those who had smoked ≥ 1 cigarette/day the past 12 months and the category “non-smokers” included those who had never smoked and those who had stopped smoking for more than 12 months.

### 2.4. Follow-Up Evaluation

In 2022, 2169 out of 3042 participants were found and had agreed to participate in the 20-year follow-up (participation rate 71%). The number of participants that were lost because of incorrect, missing, or changed addresses or telephone numbers was 771, while 102 refused to take part in the study again. In case of death during the follow-up period, information was obtained either from medical records or from relatives.

The endpoint of the present study was the development of T2DM, defined according to the criteria of the American Diabetes Association [13]. The number of participants with complete data for the evaluation of T2DM incidence in the 20-year follow-up assessment was 2138 men and women (aged 44 ± 14 years; 18–89 years, 49% men). For the evaluation of T2DM incidence and its predictors, those who were defined as having T2DM at baseline evaluation (i.e., 138 participants) were excluded from the analyses; thus, information from 974 men (aged 43 ± 13 years; 18–89 years) and 1026 women (aged 42 ± 13 years; 18–89 years) was analyzed here.

### 2.5. Statistical Analysis

Categorical variables are presented as relative frequencies (%), and continuous variables are presented as mean values (Standard deviation (SD)). Associations between categorical characteristics were based on the Pearson chi-square test. Associations between normally distributed variables and the T2DM incidence were evaluated through the independent samples *t*-test, while their association with the trajectories of participants’ adherence level to the Mediterranean diet was examined with the one-way Analysis Of Variance (ANOVA). Whether these variables were normally distributed was tested through P–P plots, and equality of variances was tested through Levene’s test. Bonferroni correction was applied in the case of multiple comparisons. The main endpoint of the study was the 20-year incidence of T2DM and it was calculated as the ratio of new cases to the total number of participants in the 20-year follow-up (*n* = 2000). Risk ratios (RRs), as a proxy of Odds Ratios (ORs) and their corresponding 95% Confidence Intervals (95% CIs) for the trajectories of participants’ adherence level to the Mediterranean diet in relation to the examined endpoint within the 20-year follow-up period, were evaluated through multivariable logistic regression analysis. Four models were created and adjusted for several potential confounders; i.e., a crude model that contained only the trajectories; a model adjusted for age and sex; a model adjusted for age, sex, years of education, current smoking, total energy intake, physical activity, and family history of T2DM; and the full model also incorporating anthropometric indices. The Hosmer–Lemeshow statistic was calculated to evaluate the models’ goodness-of-fit. STATA software, version 17 (MP & Associates, Sparta, Greece), was used for all statistical analyses. The two-sided level of significance was set at *p* < 0.05.

## 3. Results

### 3.1. Incidence of Type II Diabetes Mellitus at 20-Year Follow-Up

In Table 1, the 20-year incidence of T2DM is presented, stratified by participants’ sex and age. During the follow-up period, 526 new cases of T2DM were observed, i.e., a total incidence rate of 26.3% (95%CI 24.4%, 28.3%), of which 306 were men (incidence rate 31.4%) and 220 were women (incidence rate 21.4%) (*p* for gender difference < 0.001). A significant trend in T2DM incidence was observed across age groups in both sexes. The highest T2DM incidence was observed in the 56–65 age group (33.3%), followed by the 46–55 age group (32.5%), the 36–45 age group (26.1%), and the ≤35 age group (18.6%). A sex-related association was also revealed regarding T2DM incidence; in particular, men had higher incidence as compared to women across all age groups (*p* for gender difference < 0.001), except for the 66+ age group, where women had higher incidence as compared to men. Overall, the age–sex adjusted T2DM incidence men-to-women ratio was 1.5 (*p* < 0.001).

### 3.2. Participants’ Baseline Characteristics and 20-Year Incidence of Type II Diabetes Mellitus

Regarding lifestyle, clinical, and biochemical characteristics, as presented in Table 2, individuals who developed T2DM had increased values of BMI, waist circumference, waist-to-hip ratio, and waist-to-height ratio as compared to the rest (all *p*-values < 0.001). In addition, individuals who developed T2DM had much higher cholesterol levels (in terms of total cholesterol, LDL cholesterol, non-HDL cholesterol, and apoB100) and triglycerides levels (all *p*-values < 0.001). Adherence to the Mediterranean diet, along with total energy intake, were significantly lower among those who developed T2DM during the follow-up period. Moreover, the prevalences of hypertension and hypercholesterolemia were higher among those who developed T2DM as compared to those who did not (all *p*-values < 0.001).

### 3.3. Participants’ Baseline Characteristics and Trajectories of Adherence to the Mediterranean Diet

Four trajectories regarding the adherence to the Mediterranean diet were formed as explained in the Section 2 (Table 3). It was observed that from baseline to 10 years, 418 participants (20.9%) were classified under the “Sustained low” trajectory, 158 (7.9%) under the “Increasing” trajectory, 978 (48.9%) under the “Decreasing” trajectory, and 446 (22.3%) under the “Sustained high” trajectory. Long-term adherence to the Mediterranean diet was associated with younger age (*p* < 0.001) and women (*p* < 0.001), as well as lower values of anthropometric indices, i.e., BMI, waist circumference, waist-to-hip ratio, and waist-to-height ratio (all *p*-values < 0.001). Participants in the “Sustained high” trajectory exhibited significantly lower energy intake compared to participants in the other trajectories (*p* < 0.001). Moreover, sustained adherence to the Mediterranean diet was associated with a lower prevalence of medical history of hypertension and hypercholesterolemia (all *p*-values < 0.001). Finally, regarding lipidemic profile, participants in the “Sustained high” trajectory demonstrated better lipid profiles, characterized by lower levels of total-cholesterol, LDL cholesterol, triglycerides, non-HDL cholesterol as well as apoB100 (all *p*-values < 0.001), and higher levels of HDL cholesterol (*p* < 0.001). Concerning glycemic profile, fasting glucose, insulin level, and HOMA-IR were significantly lower among participants in the “Sustained high” trajectory as compared to the rest trajectories (*p* < 0.001); subjects in the “Sustained high” trajectory exhibited significantly decreased values of inflammatory biomarkers, like C-reactive protein, interleukin-6, and tumor necrosis factor-alpha. Individuals consistently close to the Mediterranean diet throughout the studied period had improved glycemic, lipidemic, as well as inflammatory profiles (at baseline and at 10-y follow-up) (all *p*-values < 0.001).

From the unadjusted analyses, it seems that adherence to the Mediterranean diet was associated with lower T2DM incidence. However, residual confounding effects may exist. Thus, multi-adjusted analyses were applied (Table 4). Long-term high adherence to the Mediterranean diet was highly protective against the onset of T2DM as compared to the sustained low adherence to the Mediterranean diet. The protective association of the “Sustained high” adherence level to the Mediterranean diet remained significant in all models, i.e., crude model [RR = 0.43 (95%CI 0.32, 0.60); *p* = 0.045], age- and sex-adjusted model [RR = 0.58 (95%CI 0.40, 0.85); *p* = 0.037], further-adjusted models (age, sex, years of education, current smoking, total energy intake, physical activity, family history of T2DM) [RR = 0.59 (95%CI 0.45, 0.93); *p* = 0.026]; and the fully adjusted model including BMI [RR = 0.79 (95%CI 0.43, 0.92); *p* = 0.032]; BMI was retained in the full model as it showed higher goodness-of-fit compared to the other anthropometric factors.

## 4. Discussion

In the present study, the longitudinal association of adherence to the Mediterranean diet with the 20-year T2DM incidence was examined in a representative sample of Greek adults. In the crude analysis, individuals who consistently adhered to the Mediterranean diet throughout the follow-up period exhibited a significant decrease in 20-year T2DM incidence by 57% compared to those who continuously strayed away from the traditional dietary pattern, underlying the significant protective effect of long-term adherence to the Mediterranean diet on diabetes development. The observed beneficial association remained significant regardless of the moderating or mediating effect of various potential confounders, suggesting the strong influence of longitudinal adherence to the Mediterranean diet on T2DM risk. Furthermore, it seems that even in participants who strayed away from the traditional Mediterranean dietary pattern, a protection against T2DM risk remained, whereas individuals who strayed away from the Mediterranean dietary pattern throughout the follow-up period experienced the highest 20-year T2DM risk.

Among various healthy dietary patterns, the Mediterranean diet has received great attention in terms of chronic disease prevention and treatment. The Mediterranean diet refers to the traditional dietary habits adopted in the Mediterranean region in mid 1950s and 1960s and includes olive oil as the main source of dietary fat; a wide variety of plant-based foods, like fruits, vegetables, whole grains, legumes, and nuts; moderate amounts of full-fat dairy products; moderate amounts of fish and poultry, in combination with decreased amounts of red meat; and a low consumption of wine, usually accompanying meals [7]. A variety of observational studies and clinical trials have already shown the beneficial effects of the Mediterranean dietary pattern on cardiometabolic diseases, some types of cancer, and neurological disorders [7,8,19,20,21,22]. According to the findings from a meta-analysis including 248,140 participants with a mean follow-up of 10.8 years, individuals with high adherence to the Mediterranean diet demonstrated a 11% decreased risk of developing T2DM compared to those with low adherence. Similarly, in studies where the follow-up was longer than 10 years, the T2DM risk was also decreased by 10–12%, whereas in studies where the follow-up was sorter, no difference between groups with different levels of adherence was found [23]. A large, three-arm randomized clinical trial, i.e., Prevencion con Dieta Mediterranea study (PREDIMED), showed that the reduction in 5-year T2DM incidence was 52% in the two groups who adhered to the Mediterranean diet, either via a supplement of virgin olive oil or mixed nuts, compared with the control group, who followed a low-fat diet, regardless of changes in body weight or physical activity [24]. However, few studies have evaluated lifelong adherence to the Mediterranean diet on health outcomes. According to findings from a systematic review of prospective studies, plant-based dietary patterns characterized by legumes, fruits, fish, poultry, and vegetables were associated with a decreased risk of T2DM incidence by 16%, while the Mediterranean diet seemed to be protective against T2DM risk by 13% [20].

The prevalence of T2DM is increasing predominantly due to increased adiposity, whose proxies are BMI and anthropometric indices, such as waist circumference, hip circumference, waist-to-hip ratio, and waist-to-height ratio. Specifically, according to findings from an exposure-wide umbrella review of meta-analyses, the increasing values of BMI were significantly associated with a 6.88-times-increased risk of T2DM [21]. Furthermore, increased BMI, waist circumference, waist-to-hip ratio, and waist-to-height ratio are indicative of elevated intra-abdominal visceral fat, which in turn disrupts insulin metabolism through the release of serum free fatty acids [25], which is consistent with the findings from the present study. The anthropometric characteristics were also associated with increased 20-year T2DM risk in the present analysis of the ATTICA study, but this seemed to be modified among participants who sustained high adherence to the Mediterranean diet.

Aging also plays a significant role in the development of T2DM. Based on the findings of the present study, the 20-year T2DM incidence was significantly elevated with increasing age, confirming that age is a major risk factor for T2DM development. Many investigators have associated aging with increasing body weight and visceral fat accumulation. However, as has been observed, lowering the rates of obesity will not eliminate the incidence of T2DM, especially as the average life expectancy increases [26]. T2DM is a heterogeneous syndrome, suggesting that aging augments T2DM risk through pathophysiological mechanisms independent of obesity [27]. Moreover, based on an extensive review regarding the pathophysiology of type Ι and type ΙΙ diabetes mellitus, insulin resistance exhibits a major role in the development of a cluster of cardiometabolic disorders, including dyslipidemia, inflammation, and high blood pressure [28]. Consistent with our findings, the incidence of T2DM significantly increases among individuals with a family history of both hypertension and hypercholesterolemia, as well as with increased levels of inflammatory biomarkers, i.e., C-reactive protein and interleukin-6, as reported by others [28].

Furthermore, sex is considered a major risk factor for the development of T2DM, i.e., men exhibit a higher T2DM incidence compared to women in the case of either young or middle-aged populations, which is consistent with the findings of our present study [1]. Notably, the man-to-woman T2DM incidence ratio was consistently above 1 across all age groups, except for the 66+ age group, indicating that the increasing trend was reversed. A possible explanation of this reverse trend could be attributed to hormonal changes in postmenopausal women, i.e., the decreased production of estrogens, which is associated with elevated risk of cardiometabolic diseases and increased insulin resistance [29]. In fact, a reversible trend was observed in the 66+ age group and not in the 46–55 age group or the 56–65 age group, even though the majority of women experience menopause between 45 and 56 years old as a natural part of biological ageing [30]; this may imply the crucial role of ageing concerning estrogen production, a metabolic process which, in turn, is associated with decreased insulin sensitivity [29].

The holistic approach of diet, through dietary patterns, is an alternative method to traditional individual nutrient or food analysis because it can assess the cumulative effects of the overall diet given the synergistic or antagonistic properties of foods. Individuals consume a variety of foods in different combinations and the approach of single foods or nutrients does not, therefore, reflect the reality [31]. Several healthy, plant-based dietary patterns have received great attention in terms of non-communicable disease prevention and treatment, including cardiometabolic disorders and diabetes [32], whereas Mediterranean-style and low-carbohydrate eating plans seem to be appropriate for individuals with prediabetes [33,34,35,36,37]. The protective effect of the Mediterranean diet, specifically concerning the prevention of T2DM, has been examined in detail the past years through randomized controlled trials as well as cross-sectional and prospective studies, highlighting the ability of this diet to prevent the onset of T2DM through the management of glucose and insulin levels, body weight, and the inflammation process [9,38,39]. The possible biological mechanisms explaining these observations include beneficial effects on all major T2DM risk factors, such as insulin resistance, chronic inflammation, and oxidative stress [40]. High concentrations of antioxidants [41], magnesium [42], cereal fiber [43], and bioactive compounds found in wine, such as polyphenols and resveratrol [44], in combination with weight control [45] are the major parameters that may contribute to reduced T2DM risk, mainly through the amelioration of insulin and glucose signaling.

## 5. Strengths and Limitations

The ATTICA is a prospective cohort study with a big sample and a long follow-up period (i.e., 20 years). Moreover, multiple assessments were performed (at baseline, 5-, 10- and 20 years), making the evaluation of the Mediterranean diet trajectories possible. However, since our study is an observational one, residual confounding by unmeasured or unknown parameters may exist; thus, the results should be interpreted and generalized with caution. The exact date of T2DM development was not available in most of the cases (only the diagnosis date was available, limiting the opportunity for accurate incidence rate ratio calculations). The evaluation of the longitudinal trends of trajectories of the Mediterranean diet adherence in association with 20-year T2DM incidence can reflect the long-term Mediterranean diet’s impact, but misreporting of dietary and lifestyle habits may exist as the assessment was based on food frequency questionnaires that semi-quantitively evaluated consumption of foods and beverages. Moreover, the presented findings should be replicated through other longitudinal studies with long term follow-up in different populations with a variety of ethnic, clinical, sociodemographic, and cultural characteristics in order to be generalizable for different regions around the world.

## 6. Conclusions

The present cohort study, which consisted of apparently healthy, Greek adults free of diabetes, demonstrated that a “sustained” high adherence to the Mediterranean diet, both at baseline and at follow-up, was associated with a significant protection against the onset of T2DM regardless of several traditional risk factors. This longitudinal protective effect of adherence to the Mediterranean diet proposes that the Mediterranean diet may be a useful tool for utilization by physicians and other health professionals to decrease the burden of T2DM-related morbidity and mortality.

## Figures and Tables

**Table 1:** The 20-year incidence (%) of T2DM with 95% CI, stratified by the N = 2000 ATTICA study participants’ sex and age at baseline; ATTICA study (2002–2022).

Age at Baseline	20-Year Incidence (%) of T2DM		Total (N = 2000)	*p*-Value ^1^	Men-to-Women Ratio
	Men (N = 974)	Women (N = 1026)			
≤35 y (N = 613)	22.1% (17.5, 27.4)	15.8% (12.3, 20.1)	18.6% (15.7, 21.9) *	<0.001	1.4
36–45 y (N = 602)	31.3% (26.3, 36.8)	21.0% (16.8, 25.9)	26.1% (22.7, 29.7) *		1.5
46–55 y (N = 496)	37.1% (31.6, 43.0)	26.8% (21.4, 32.9)	32.5% (28.5, 36.7) *		1.4
56–65 y (N = 183)	46.7% (35.8, 57.8)	24.1% (17.0, 32.9)	33.3% (26.9, 40.4) *		1.9
66+ y (N = 106)	29.3% (19.2, 42.0)	33.3% (21.7, 47.5)	31.1% (23.1, 40.5)		0.9
Total	31.4% (28.6, 34.4) **	21.4% (19.0, 24.1) **	26.3% (24.4, 28.3)		1.5
*p*-value ^2^	<0.001				

*p*-value was based on the Pearson chi-square test; ^1^ *p*-value refers to the comparison among the 5 age groups; ^2^ *p*-value refers to the comparison between men and women; CI = Confidence Interval based on 1-sample proportion test without continuity correction; * denotes the statistically significant difference between men and women in each age category (Pearson chi-square test); ** denotes the significant difference among the age groups in each sex (Pearson chi-square test); Bonferroni correction was applied for multiple comparisons. Abbreviations: T2DM: type II diabetes mellitus; y: years old.

**Table 2:** Baseline sociodemographic, anthropometric, lifestyle, clinical, and biochemical characteristics of the N = 2000 ATTICA study participants, both in total and stratified, according to the 20-year incidence of T2DM; ATTICA study (2002–2022).

	Total Sample (N = 2000)	Developed T2DM during 20-Year FU		*p*-Value
		Yes (N = 526)	No (N = 1474)	
Sociodemographic characteristics				
Age; years, Mean (SD)	42.7 (12.9)	45.2 (12.6)	41.8 (12.9)	<0.001
Sex; % men	48.7	58.2	45.3	<0.001
Years of school; Median (IQR)	12 (12–16)	12 (12–16)	12 (12–16)	0.079
Anthropometric characteristics				
Body Mass Index; kg/m^2^, Mean (SD)	26 (4.4)	27 (4.6)	25.6 (4.3)	<0.001
Waist circumference; cm, Mean (SD)	89.1 (15.1)	93.1 (13.9)	87.7 (15.3)	<0.001
Waist-to-hip ratio; units, Mean (SD)	0.9 (0.1)	0.9 (0.1)	0.8 (0.1)	<0.001
Waist-to-height ratio; units, Mean (SD)	0.5 (0.1)	0.5 (0.1)	0.5 (0.1)	<0.001
Lifestyle characteristics				
MedDietScore; units (0–55), Median (IQR)	27 (25–28)	26 (25–27)	27 (26–28)	<0.001
High level of adherence to Mediterranean diet; %	72.7	63.1	76.1	<0.001
Total energy intake; kcal, Mean (SD)	2338 (932)	2479 (978)	2283 (908)	0.006
Current smoking; % yes	43.2	42.9	43.3	0.865
Physical activity; % yes	36.4	37.1	36.1	0.688
Clinical characteristics				
Hypertension; %	27.5	35.5	24.7	<0.001
Hypercholesterolemia; %	39.2	46.9	36.5	<0.001
Family history of T2DM; %	53.4	54.5	53.0	0.901
Biochemical parameters				
Total cholesterol; mg/dL, Mean (SD)	191.9 (41.2)	202.1 (41)	188.6 (40.7)	<0.001
LDL cholesterol; mg/dL, Mean (SD)	120.7 (37.2)	128.5 (36.6)	118.1 (37)	<0.001
HDL cholesterol; mg/dL, Mean (SD)	49 (14.3)	46.4 (11.9)	49.8 (15)	<0.001
Triglycerides; mg/dL, Mean (SD)	112.3 (80.2)	130.2 (108.7)	106.6 (67.7)	<0.001
Non-HDL cholesterol; mg/dL, Mean (SD)	142.6 (43.1)	155.2 (42.7)	138.3 (42.4)	<0.001
ApoB100; mg/dL, Mean (SD)	106.0 (40.3)	113.6 (30.3)	103.6 (42.7)	<0.001
Fasting glucose; mg/dL, Mean (SD)	88.7 (12)	104.2 (6.4)	83.7 (8.7)	<0.001
Fasting insulin; μU/mL, Mean (SD)	12.5 (2.1)	14.2 (3)	12 (1.2)	<0.001
HOMA-IR; units, Mean (SD)	2.8 (0.7)	3.7 (0.5)	2.5 (0.5)	<0.001
C-reactive protein; mg/L, Mean (SD)	1.9 (2.4)	2.2 (2.6)	1.8 (2.3)	0.001
Interleukin-6; pg/mL, Mean (SD)	1.4 (0.5)	1.5 (0.6)	1.4 (0.5)	<0.001
Tumor necrosis factor-alpha; pg/mL, Mean (SD)	6.1 (4.6)	6.1 (4.1)	6.0 (4.7)	0.718

*p*-value was based on the Pearson chi-square test (categorical characteristics), on the independent samples *t*-test (normally distributed continuous characteristics), and on the Mann–Whitney U test (non-normally distributed continuous characteristics). Level of adherence to the Mediterranean Diet was evaluated through the 11-item MedDietScore, and participants scoring at the middle and upper tertiles of the MedDietScore were classified as having a high level of adherence. Participants’ physical activity level was assessed through the short-form International Physical Activity Questionnaire. Subjects who reported smoking ≥ 1 cigarette/day or had ceased smoking within 12 months were classified as current smokers. Abbreviations: T2DM: type II diabetes mellitus; SD: standard deviation; FU: follow-up; ApoB100: apolipoprotein B100; HOMA-IR: Homeostatic Model Assessment for Insulin Resistance.

**Table 3:** Baseline sociodemographic, anthropometric, lifestyle, clinical, and biochemical characteristics of the N = 2000 ATTICA study participants stratified by the 10-year trajectories of their adherence level to the Mediterranean diet; ATTICA study (2002–2022).

	Trajectories of Participants’ Adherence Level to the Mediterranean Diet (2002–2012)				*p*-Value
	Sustained Low Adherence Level	Increasing Adherence Level	Decreasing Adherence Level	Sustained High Adherence Level	
No. participants	418 (21%)	158 (8%)	978 (49%)	446 (22%)	
Sociodemographic characteristics					
Age; years, Mean (SD)	51.6 (13.6)	51.9 (10.4)	41.8 (11.3)	34.9 (9.7)	<0.001
Sex; % men	77.3	82.9	46.0	15.4	<0.001
Years of school; Median (IQR)	12 (6–16)	12 (12–16)	12 (12–16)	12 (12–16)	<0.001
Anthropometric characteristics					
Body Mass Index; kg/m^2^, Mean (SD)	29.5 (4.6)	30.1 (4.1)	25.5 (3.4)	22.5 (2.5)	<0.001
Waist circumference; cm, Mean (SD)	100.8 (13.4)	102.1 (11)	87.7 (12.9)	77.3 (10.1)	<0.001
Waist-to-hip ratio; units, Mean (SD)	0.9 (0.1)	0.9 (0.1)	0.8 (0.1)	0.8 (0.1)	<0.001
Waist-to-height ratio; units, Mean (SD)	0.6 (0.1)	0.6 (0.1)	0.5 (0.1)	0.5 (0.1)	<0.001
Lifestyle characteristics					
MedDietScore; units (0–55), Median (IQR)	20 (15–25)	25 (24–25)	27 (26–28)	28 (28–29)	<0.001
Total energy intake; kcal, Mean (SD)	2498 (979)	2392 (878)	2424 (951)	2109 (879)	<0.001
Current smoking; % yes	39.2	42.1	43.0	45.2	0.358
Physical activity; % yes	36.7	40.8	34.5	37.1	0.431
Clinical characteristics					
Hypertension; %	48.5	45.8	25.2	8.9	<0.001
Hypercholesterolemia; %	50.6	51.3	40.2	25.9	<0.001
Family history T2DM; %	51.1	57.1	51.5	54.3	0.916
Biochemical parameters					
Total cholesterol; mg/dL, Mean (SD)	204.6 (42)	200.2 (34.8)	193 (41.9)	178.4 (36.5)	<0.001
LDL cholesterol; mg/dL, Mean (SD)	131.2 (39.1)	128 (33.9)	121.6 (37.3)	109.4 (33.1)	<0.001
HDL cholesterol; mg/dL, Mean (SD)	45.1 (14)	43.4 (10.4)	49.6 (13.9)	53.5 (15.5)	<0.001
Triglycerides; mg/dL, Mean (SD)	143.3 (118.7)	141.5 (86.9)	110.5 (67.6)	80.9 (38.3)	<0.001
Non-HDL cholesterol; mg/dL, Mean (SD)	162.0 (45.2)	159.0 (37.8)	143.5 (43.1)	125.3 (38.7)	<0.001
ApoB100; mg/dL, Mean (SD)	120.6 (46.6)	119.0 (28.0)	106.6 (38.0)	92.0 (28.9)	<0.001
Fasting glucose; mg/dL, Mean (SD)	91.4 (12.8)	94.1 (11.4)	88.2 (11.7)	86 (11.3)	<0.001
Fasting insulin; μU/mL, Mean (SD)	13.6 (3.2)	13.7 (1.3)	12.4 (1.5)	11.5 (1.4)	<0.001
HOMA-IR; units, Mean (SD)	3.7 (2.5)	4.1 (3.3)	2.9 (1.5)	2.5 (0.9)	<0.001
C-reactive protein; mg/L, Mean (SD)	2.5 (2.8)	2.3 (2.4)	1.8 (2.2)	1.4 (2.2)	<0.001
Interleukin-6; pg/mL, Mean (SD)	1.7 (0.6)	1.6 (0.4)	1.4 (0.5)	1.3 (0.5)	<0.001
Tumor necrosis factor-alpha; pg/mL, Mean (SD)	8.6 (5.4)	8.4 (4.0)	5.8 (4.4)	4.7 (5.1)	<0.001

*p*-value was based on the Pearson chi-square test (categorical characteristics), on the one-way ANOVA (normally distributed continuous characteristics) ,and on the Kruskal–Wallis test (non-normally distributed continuous characteristics). Level of adherence to the MD was evaluated through the 11-item MedDietScore, and participants scoring at the middle and upper tertiles of the MedDietScore were classified as having a high level of adherence. Participants’ physical activity level was assessed through the short-form International Physical Activity Questionnaire. Subjects who reported smoking ≥ 1 cigarette/day or had ceased smoking within 12 months were classified as current smokers. Abbreviations: SD: standard deviation; T2DM: type II diabetes mellitus; ApoB100: apolipoprotein B100; HOMA-IR: Homeostatic Model Assessment for Insulin Resistance.

**Table 4:** Multi-adjusted logistic regression models evaluating the association between trajectories of adherence level to the Mediterranean diet and 20-year incidence of type II diabetes mellitus; the ATTICA study (2002–2022).

	Crude Model	Age-, Sex-Adjusted Model	Adjusted Model without BMI *	Fully Adjusted Model with BMI
	**RR (95%CI)**	**RR (95%CI)**	**RR (95%CI)**	**RR (95%CI)**
Increasing vs. Sustained low adherence level	1.22 (0.83–1.79)	1.19 (0.84–1.69)	1.23 (0.72–2.05)	1.24 (0.74–2.06)
Decreasing vs. Sustained low adherence level	**0.62** **(0.48–0.80)**	**0.69** **(0.53–0.89)**	**0.72** **(0.49–0.92)**	0.96 (0.65–1.88)
Sustained high vs. Sustained low adherence level	**0.43** **(0.32–0.60)**	**0.58** **(0.40–0.85)**	**0.59** **(0.45–0.93)**	**0.79** **(0.43–0.92)**

RRs and their corresponding 95%CIs were obtained through logistic regression analysis as proxy of Odds Ratios. * Adjusted model included age (years), sex (men vs. women), years of education, smoking (yes vs. no), total energy intake (kcal), physical activity (yes/no), and family history of T2DM (yes vs. no). Reference category of trajectories of adherence level to the Mediterranean diet: sustained low adherence level. Level of adherence to the Mediterranean diet was evaluated through the MedDietScore. RR (95%CI) in bold denote the statistically significant results. Abbreviations: BMI: Body mass index; RR: Relative Risk; CI: Confidence Interval.

## Data Availability

The data presented in this study are available on request from the corresponding author. The data are not publicly available due to privacy restrictions.
